# Neonates with coronavirus disease 2019 acquired from infected mothers: the incompatibility of maternal intensity and infant lung involvement: two case reports

**DOI:** 10.1186/s13256-021-02698-5

**Published:** 2021-05-28

**Authors:** Somayeh Moeindarbary, Azam Pourhoseini, Parvaneh Layegh, Zahra Shahriari, Faezeh Fayyaz, Milad Bahrami, Mahdi Rafiee

**Affiliations:** 1 Department of Obstetrics and Gynecology, Neonatal and Maternal Research Center, Faculty of Medicine, Mashhad University of Medical Sciences, Mashhad, Iran; 2 Department of Radiology, School of Medicine, Mashhad University of Medical Sciences, Mashhad, Iran; 3 Student Research Committee, Faculty of Paramedical sciences, Mashhad University of Medical Sciences, Mashhad, Iran

**Keywords:** Vertical transmission, COVID-19, Case report, Neonates, Maternal–fetal transmission

## Abstract

**Background:**

The outbreak of coronavirus disease 2019 (COVID-19) caused by the novel severe acute respiratory syndrome coronavirus 2 (SARS-CoV-2) was declared a public health emergency by the World Health Organization on January 30, 2020. The results of recent studies have suggested that neonates may present symptoms of COVID-19. Although the presentation of the disease in neonates is known to vary, only a limited number of studies have investigated newborns infected with COVID-19.

**Case presentation:**

This study presents two Asian cases of newborns with COVID-19. Maternal–fetal or postnatal transmission was suggested based on the simultaneity of maternal infection. Chest radiography in one of the neonates showed severe lung involvement. Despite support and resuscitation attempts, the poor clinical condition of the neonate led to his death. However, the two mothers and one of the neonates were discharged from the hospital in good general condition.

**Conclusion:**

The neonates had worse clinical conditions than the mothers, and the intensity of pneumonia and level of lung involvement in the newborns were not associated with the stage and severity of the disease in the mothers with COVID-19.

## Background

Severe acute respiratory syndrome coronavirus 2 (SARS-CoV-2) is the third coronavirus of the twenty-first century to become a global concern. This virus causes a new viral respiratory disease, named coronavirus disease 2019 (COVID-19) by the World Health Organization [1]. More than 13.8 million cases have been reported across 188 countries and territories since July 17, 2020, resulting in more than 592,000 deaths. Among the affected population, more than 7.77 million cases have recovered [2]. According to the notice issued from the People's Republic of China on February 2, 2020, newborns can also be infected with SARS-CoV-2 due to the immaturity of their immune systems [3]. Although the epidemic continues to progress, our knowledge about pediatric COVID-19 infections and their clinical implications remains scarce [4].

Recent reports have suggested that the disease is generally less severe in children; however, fatal cases have been reported [4, 5]. Children constitute a small proportion of reported cases, with about 1% and 4% of cases occurring in children under the age of 10 and in those 10–19 years of age, respectively [6]. It is not clear whether children are less susceptible to the infection or are simply less symptomatic. They are likely to have milder symptoms and a lower chance of severe disease than adults. Indeed, age and pre-existing and underlying conditions are associated with increased severity [6, 7]. Nevertheless, the presentation of the disease is variable in children, and sometimes requires aggressive management [7, 8].

Many studies have revealed the signs and symptoms of infected adults with SARS-CoV-2. Although some articles have discussed COVID-19 complications in children, the existing information on the pediatric population infected with COVID-19 is scarce [2, 9]. Pregnant women may be at higher risk of severe COVID-19 infection based on reported results from similar viruses, such as severe acute respiratory syndrome (SARS) and Middle East respiratory syndrome (MERS); nonetheless, data for COVID-19 are inadequate [11]. To the best of our knowledge, few studies have been dedicated to investigating the clinical features and vertical transmission of SARS-CoV-2 in pregnant women. Therefore, the question remains whether SARS-CoV-2 can be transmitted during pregnancy and cause serious infection for newborns. [10–12]. This study reports two cases of neonates infected with COVID-19 who were born from infected mothers with different related symptoms.

## Case presentation

### Case 1

A 35-year-old Asian woman, gravida 4, para 3, with gestational age of 39 weeks and a history of three previous cesarean sections was referred to the Imam Reza Hospital, Mashhad, Iran, due to uterine contractions. She had no history of underlying diseases and no contact with COVID-19-infected cases. She had no fever or symptoms such as cough, sore throat, or muscle weakness on admission. She also did not complain of gastrointestinal disorders such as diarrhea and vomiting. Upon admission, her vital signs were as follows: blood pressure 110/70 mmHg, heart rate 130 beats/minute, temperature 37.5 °C, respiratory rate 26 breaths/minute, and oxygen saturation (SpO_2_) 96%.

**Fig. 1:**
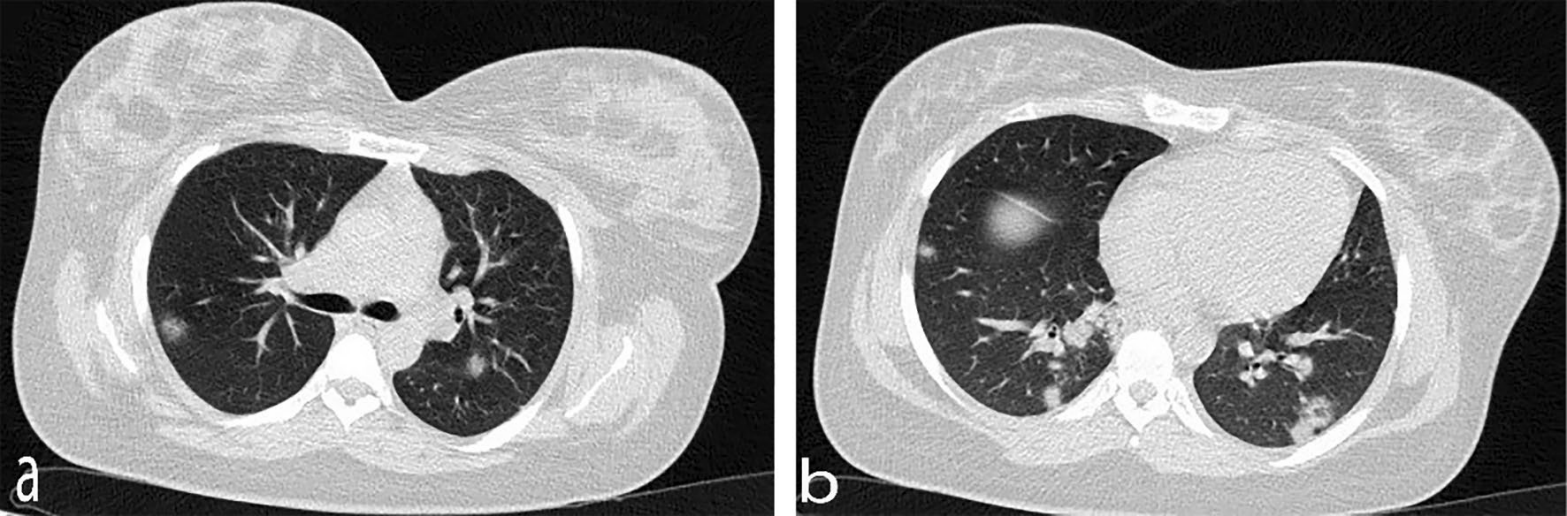
Axial chest computerized tomography showing bilateral multifocal rounded consolidations with surrounding ground-glass opacities

**Fig. 2:**
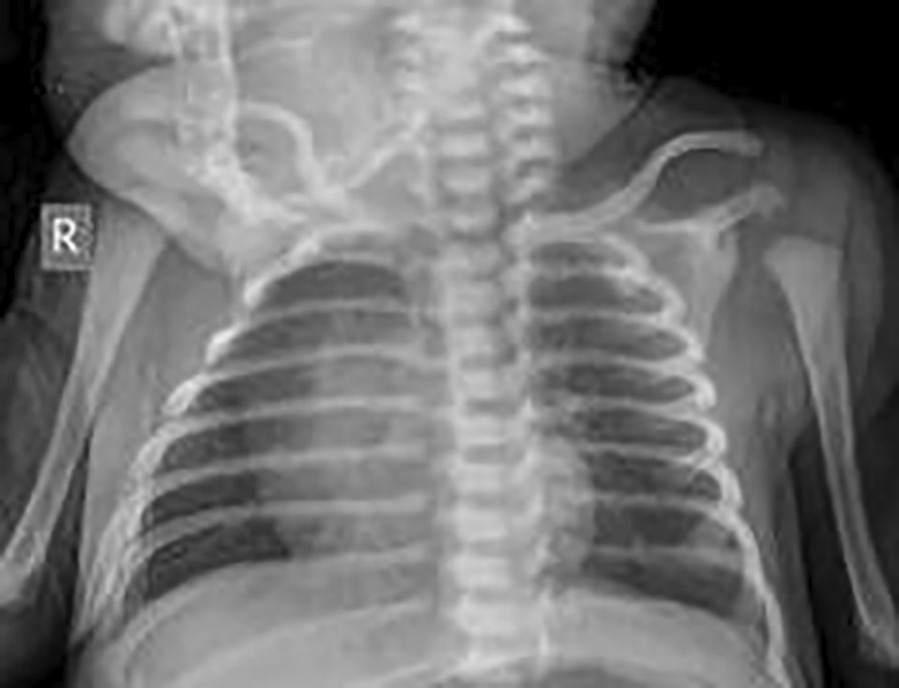
Anteroposterior chest X-ray view of term newborn showing no abnormal opacity in either lung and overall normal lungs

According to the laboratory results, the lymphocyte count was lower than normal (1× 10^9^/L), and platelet count, hepatic enzymes, and creatinine level were within the normal range, while the C-reactive protein level (CRP) was significantly high (Table 1). Coagulation function and blood biochemistry were normal. Due to the high CRP level and probability of COVID-19 infection, a GeneXpert COVID-19 ribonucleic acid (RNA) reverse transcription polymerase chain reaction (RT-PCR) test was performed, which returned positive results. The results of computed tomography (CT) revealed bilateral involvement of the lungs and multifocal rounded consolidations with surrounding ground-glass opacities (Fig. 1). The patient underwent cesarean section due to the onset of uterine contractions and a history of previous cesarean sections. A full-term female neonate was born with a birth weight of > 3000 g and 1- and 5-minute APGAR [appearance, pulse, grimace, activity, respiration] scores of 8 and 9, respectively. A throat swab sample was tested within 24 hours after birth, the positive result of which confirmed the neonate's infection with SARS-CoV-2. This newborn suffered from transient tachypnea and needed nasal continuous positive airway pressure. The results of chest CT showed no abnormal opacities (Fig. 2). The neonate had normal breathing 3 days after birth and was discharged from the neonatal intensive care unit (NICU) 6 days later. The mother was also discharged in good general condition 1 week after delivery.

**Table 1:** Clinical and laboratory characteristics of mothers

Signs and symptoms	Case 1	Case 2
Fever	No	Yes
Cough	No	Yes
Fatigue	No	Yes
Headache	No	No
Dyspnea	Yes	No
Heart rate (per minute, at arrival)	130	110
White blood cell count (×10^9^/L)	17.2	9.2
Lymphocyte count (×10^9^/L)	1.0	1.0
Hemoglobin (g/L)	12.8	11
Platelet count (×10^9^/L)	158	120
C-reactive protein (mg/L)	79	50
Alanine aminotransferase (U/L)	15	30
Aspartate aminotransferase (U/L)	25	35
Creatinine (μmol/L)	0.6	0.8

### Case 2

**Fig. 3:**
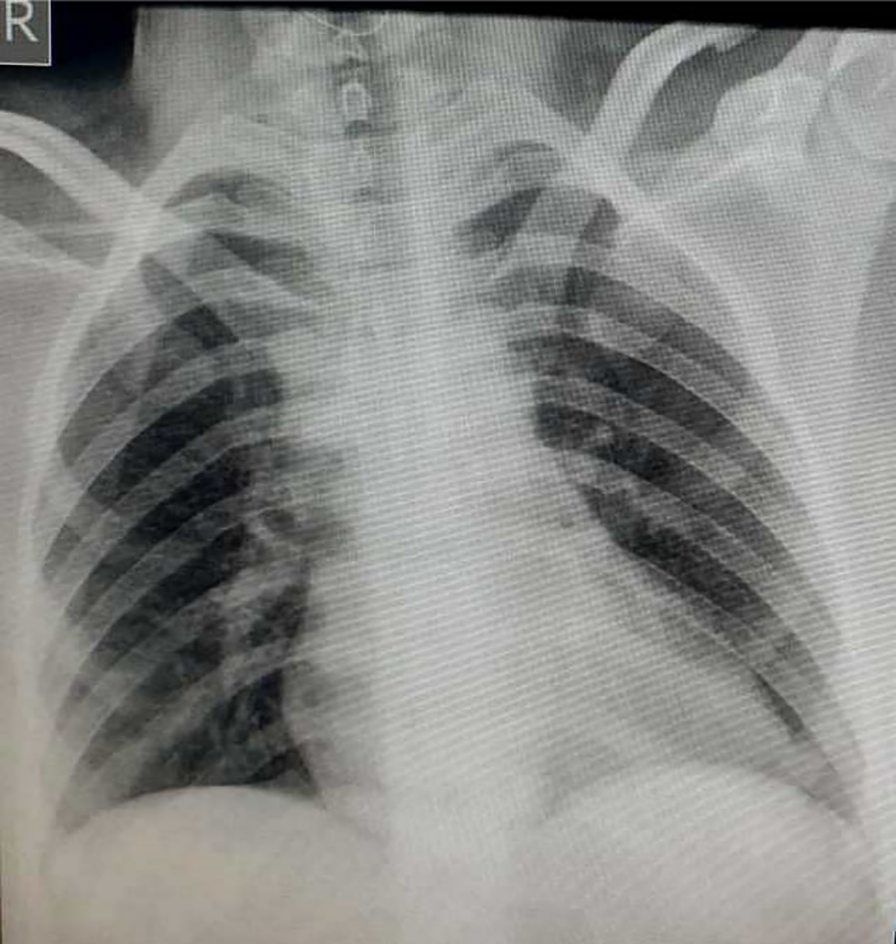
Posteroanterior chest X-ray view with normal lung appearance

**Fig. 4:**
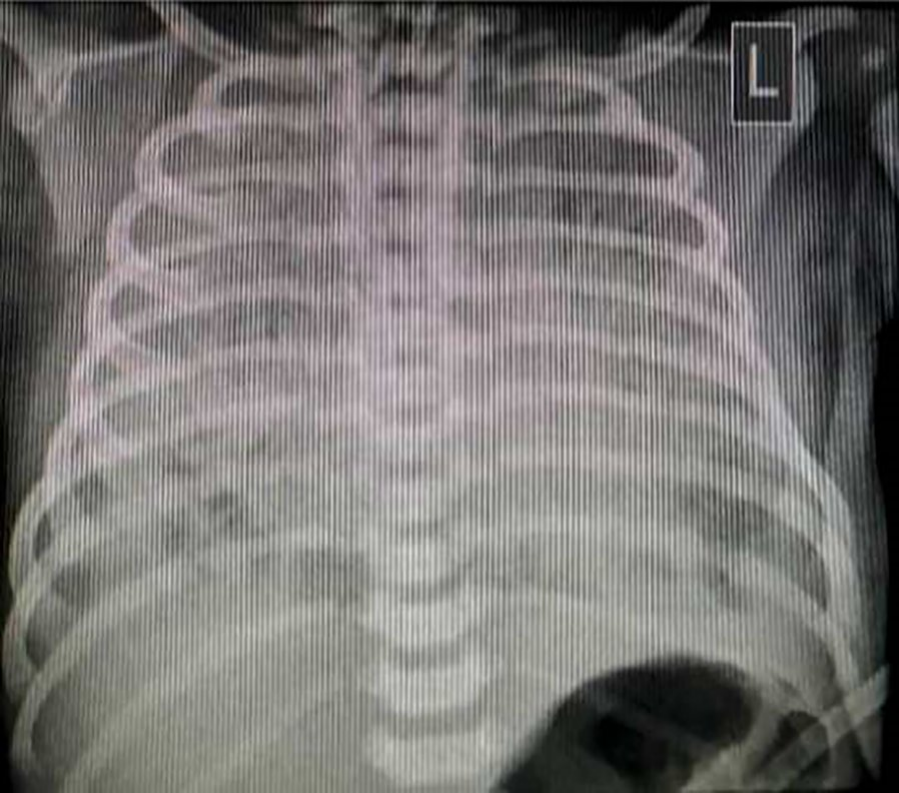
Plain radiograph of posteroanterior view in supine position, showing diffuse ground-glass opacity in both lungs

A 31-year-old Asian woman, gravida 2, para 1, with gestational age of 39 weeks and a history of cesarean section was referred to the Imam Reza Hospital due to increased uterine contractions. She had no underlying diseases. Her vital signs were recorded as follows: blood pressure 120/70 mmHg, SpO_2_ 96%, heart rate 110 beats/minute, temperature 39°C, and respiratory rate 26 breaths/minute. She had experienced coughing and sore throat during the previous week and complained of muscle weakness. The patient mentioned that her husband had also experienced the same symptoms. Laboratory tests showed an increased level of CRP and lymphopenia (1.0 × 10^9^/L) (Table 1). Chest X-ray and RT-PCR test were performed in the first 24 hours due to the probability of COVID-19 infection. Lung appearance was normal, with no signs of typical viral pneumonia (Fig. 3). COVID-19 test was conducted and the positive result of RT-PCR assay confirmed SARS-CoV-2 infection. The patient underwent a cesarean section due to the onset of uterine contractions and a history of previous cesarean section. A male neonate was born with a birth weight of 3400 g and 1- and 5-minute Apgar scores of 2 and 6, respectively. The neonate was intubated due to severe respiratory distress and transferred to the NICU. The result of arterial blood gas showed a pH of 6.86, partial pressure of carbon dioxide (pCO_2_) of 97 mmHg, partial pressure of oxygen (pO_2_) of 49 mmHg, HCO_3_ of 16 mEq/L, and a base excess of −18.5 mmol/L. Echocardiography and chest radiography were performed due to decreased oxygen saturation and increased respiratory distress. The echocardiogram result was normal; however, chest radiography suggested severe lung involvement (Fig. 4). The positive result of COVID-19 testing confirmed that the newborn was infected with SARS-CoV-2. Despite support and resuscitation attempts, the neonate died. The mother was discharged in good general condition 5 days after delivery.

## Discussion and Conclusions

This study reported two cases of newborns infected with COVID-19. The neonates were born from mothers who were infected at the end of the third trimester of pregnancy. Both mothers underwent cesarean section to terminate the pregnancy due to the onset of uterine contractions and history of cesarean section. The positive results of the RT-PCR assay within the first 24 hours, which was performed on throat swab samples of the two newborns, confirmed SARS-CoV-2 infection of the newborns. Although brief abnormal changes were observed in the mother’s chest CT scans, there was extensive involvement and bilateral multifocal rounded consolidations in the lungs of one of the neonates, which eventually led to his death. A similar study reported one positive case of neonatal infection with COVID-19 36 hours after birth [13]. However, since sampling was performed 36 hours after birth, the possibility of vertical transmission or post-delivery transmission was unclear [14].

The common symptoms of the disease include fever, cough, fatigue, shortness of breath, and loss of smell and taste. While the majority of cases result in mild symptoms, some progress to acute respiratory distress syndrome, possibly precipitating cytokine storm, multi-organ failure, septic shock, and blood clots [17, 18]. The clinical manifestations of pediatric patients may be different from those of adults, such as lack of diarrhea and runny nose in children [19].

According to a literature review, people with COVID-19 can infect others through respiratory droplets [15]. The primary mode of transmission of COVID-19 among children is through family gatherings. However, there are limited data about intrauterine vertical transmission, and SARS-CoV-2 has not been detected in placental and fetal membrane samples [3, 16]. Alzamora *et al*. reported a neonate born from a mother infected with COVID-19, who underwent cesarean delivery. Neonatal isolation was implemented immediately after birth, without delayed cord clamping or skin-to-skin contact. Moreover, the neonate was placed in the NICU with no other COVID-19 patients. The nasopharyngeal swab of the neonate was positive for SARS-CoV-2 RT-PCR 16 hours after birth, while it was negative for virus-specific immunoglobulin M and immunoglobulin G antibodies [17]. The findings of previous studies on vaginal swab samples of pregnant women infected with human coronavirus showed the possibility of maternal transmission of this virus [18].

Contamination of the operating room or medical staff might have influenced the positive result obtained in the current study, even though the researchers attempted to reduce the spread of respiratory droplets and aerosols. Previous reports have suggested that SARS-associated coronavirus and other respiratory pathogens, including influenza, are associated with various maternal morbidities, such as spontaneous abortion, severe maternal infection, or maternal death [19, 20]. According to the results of some studies, physiological and biochemical changes and immunosuppressive conditions during pregnancy can decrease inflammatory immune responses. Therefore, pregnant women are exposed to numerous respiratory pathogens; however, these changes are necessary for the continuation of pregnancy [21, 22].

In conclusion, the intensity of pneumonia and level of lung involvement of the neonates were not associated with the stage and disease severity of the mothers infected with COVID-19.

## Data Availability

The patient information and medical records used for the case report are available from the corresponding author upon request.

## References

[1] Dong Y, Mo X, Hu Y, Qi X, Jiang F, Jiang Z,* et al*. Epidemiology of COVID-19 Among Children in China. Pediatrics. 2020. 10.1542/peds.2020-0702 32179660

[2] Li Q, Guan X, Wu P, Wang X, Zhou L, Tong Y,* et al*. Early transmission dynamics in Wuhan, China, of novel coronavirus-infected pneumonia. N Engl J Med. 2020;382(13):1199–207. 10.1056/NEJMoa2001316 PMC7121484 31995857

[3] She J, Liu L, Liu W. COVID‐19 epidemic: disease characteristics in children. J Med Virol. 2020. 10.1002/jmv.25807 PMC7228385 32232980

[4] Liu W, Zhang Q, Chen J, Xiang R, Song H, Shu S,* et al*. Detection of Covid-19 in Children in Early January 2020 in Wuhan, China. N Engl J Med. 2020;382(14):1370–1. 10.1056/NEJMc2003717 PMC7121643 32163697

[5] Coronavirus Disease 2019 in Children - United States, February 12-April 2, 2020. MMWR Morbidity and mortality weekly report. 2020;69(14):422-6. 10.15585/mmwr.mm6914e4 PMC7147903 32271728

[6] Xia W, Shao J, Guo Y, Peng X, Li Z, Hu D. Clinical and CT features in pediatric patients with COVID-19 infection: different points from adults. Pediatr Pulmonol. 2020;55(5):1169–74. 10.1002/ppul.24718 PMC7168071 32134205

[7] Zhou F, Yu T, Du R, Fan G, Liu Y, Liu Z,* et al*. Clinical course and risk factors for mortality of adult inpatients with COVID-19 in Wuhan, China: a retrospective cohort study. Lancet (London, England). 2020;395(10229):1054–62. 10.1016/S0140-6736(20)30566-3 PMC7270627 32171076

[8] Ashour HM, Elkhatib WF, Rahman MM, Elshabrawy HA. Insights into the Recent 2019 Novel Coronavirus (SARS-CoV-2) in Light of Past Human Coronavirus Outbreaks. Pathogens (Basel, Switzerland). 2020;9:3. 10.3390/pathogens9030186 PMC7157630 32143502

[9] Fu L, Wang B, Yuan T, Chen X, Ao Y, Fitzpatrick T,* et al*. Clinical characteristics of coronavirus disease 2019 (COVID-19) in China: a systematic review and meta-analysis. J Infect. 2020. 10.1016/j.jinf.2020.03.041 PMC7151416 32283155

[10] Chen Y, Peng H, Wang L, Zhao Y, Zeng L, Gao H,* et al*. Infants Born to Mothers With a New Coronavirus (COVID-19). Frontiers in Pediatrics. 2020;8:104. 10.3389/fped.2020.00104 PMC7098456 32266184

[11] Huang C, Wang Y, Li X, Ren L, Zhao J, Hu Y,* et al*. Clinical features of patients infected with 2019 novel coronavirus in Wuhan, China. Lancet (London, England). 2020;395(10223):497–506. 10.1016/S0140-6736(20)30183-5 PMC7159299 31986264

[12] Wang SX, Wang Y, Lu YB, Li JY, Song YJ, Nyamgerelt M,* et al*. Diagnosis and treatment of novel coronavirus pneumonia based on the theory of traditional Chinese medicine. J Integr Med. 2020. 10.1016/j.joim.2020.04.001. PMC7159848 32446813

[13] Wang SS, Guo L, Chen L,* et al*. A case report of neonatal COVID-19 infection in China [J/OL]. Clin Infect Dis. 2020. 10.1093/cid/ciaa225 PMC7108144 32161941

[14] Smith V, Seo D, Warty R, Payne O, Salih M, Chin KL,* et al*. Maternal and neonatal outcomes associated with COVID-19 infection: a systematic review. PLoS ONE. 2020;15(6):e0234187. 10.1371/journal.pone.0234187 PMC7272020 32497090

[15] Chan JF-W, Yuan S, Kok K-H, To KK-W, Chu H, Yang J,* et al*. A familial cluster of pneumonia associated with the 2019 novel coronavirus indicating person-to-person transmission: a study of a family cluster. Lancet. 2019;2020(395):514. 10.1016/S0140-6736(20)30154-9 PMC7159286 31986261

[16] Penfield CA, Brubaker SG, Limaye MA, Lighter J, Ratner AJ, Thomas KM,* et al*. Detection of SARS-COV-2 in Placental and Fetal Membrane Samples. Am J Obstetr Gynecol. 2020;1:100133. 10.1016/j.ajogmf.2020.100133 PMC7205635 32391518

[17] Alzamora MC, Paredes T, Caceres D, Webb CM, Valdez LM, La Rosa M. Severe COVID-19 during pregnancy and possible vertical transmission. Am J Perinatol. 2020. 10.1055/s-0040-1710050 PMC7356080 32305046

[18] Zhu H, Wang L, Fang C, Peng S, Zhang L, Chang G,* et al*. Clinical analysis of 10 neonates born to mothers with 2019-nCoV pneumonia. Transl Pediatrics. 2020;9(1):51–60. 10.21037/tp.2020.02.06 PMC7036645 32154135

[19] Assiri A, Abedi GR, Al Masri M, Bin Saeed A, Gerber SI, Watson JT. Middle east respiratory syndrome coronavirus infection during pregnancy: a report of 5 cases from Saudi Arabia. Clin Infect Dis. 2016;63(7):951–3. 10.1093/cid/ciw412 PMC5812010 27358348

[20] Shek CC, Ng PC, Fung GPG, Cheng FWT, Chan PKS, Peiris MJS,* et al*. Infants born to mothers with severe acute respiratory syndrome. Pediatrics. 2003;112(4):e254. 10.1542/peds.112.4.e254 14523207

[21] Robinson DP, Klein SL. Pregnancy and pregnancy-associated hormones alter immune responses and disease pathogenesis. Horm Behav. 2012;62(3):263–71. 10.1016/j.yhbeh.2012.02.023 PMC3376705 22406114

[22] Weinberger SE, Weiss ST, Cohen WR, Weiss JW, Johnson TS. Pregnancy and the lung. Am Rev Respir Dis. 1980;121(3):559–81. 10.1164/arrd.1980.121.3.559 6998334

